# Epidemiological and clinical features of SARS‐CoV‐2 infection in children during the outbreak of Omicron variant in Shanghai, March 7–31, 2022

**DOI:** 10.1111/irv.13044

**Published:** 2022-08-31

**Authors:** Xiangshi Wang, Hailing Chang, He Tian, Yanfeng Zhu, Jingjing Li, Zhongqiu Wei, Yixue Wang, Aimei Xia, Yanling Ge, Gongbao Liu, Jiehao Cai, Qirong Zhu, Xiaowen Zhai, Mei Zeng

**Affiliations:** ^1^ Department of Infectious Diseases Children's Hospital of Fudan University, National Children's Medical Center Shanghai China; ^2^ Paediatric Intensive Care Unit Children's Hospital of Fudan University, National Children's Medical Center Shanghai China; ^3^ Division of Medical Administration Children's Hospital of Fudan University, National Children's Medical Center Shanghai China; ^4^ Department of Hematology and Oncology Children's Hospital of Fudan University, National Children's Medical Center Shanghai China

**Keywords:** children, COVID‐19 vaccine, Omicron SARS‐CoV‐2 variant

## Abstract

**Objectives:**

This study aimed to understand the epidemiological and clinical characteristics of pediatric SARS‐CoV‐2 infection during the early stage of Omicron variant outbreak in Shanghai.

**Methods:**

This study included local COVID‐19 cases <18 years in Shanghai referred to the exclusively designated hospital from March 7 to March 31, 2022. Clinical data, epidemiological exposure, and COVID‐19 vaccination status were collected. Relative risks (RRs) were calculated to assess the effect of vaccination on symptomatic infection and febrile disease.

**Results:**

A total of 376 pediatric cases of COVID‐19 (median age: 6.0 ± 4.2 years) were referred to the designated hospital, including 257 (68.4%) symptomatic cases and 119 (31.6%) asymptomatic cases. Of the 307 (81.6%) children ≥3 years eligible for COVID‐19 vaccination, 110 (35.8%) received two doses of vaccines. The median interval between the completion of two‐dose vaccination and infection was 3.5 (interquartile range [IQR]: 3, 4.5) months. Compared with no vaccination, two‐dose COVID‐19 vaccination reduced the risks of symptomatic infection and febrile disease by 35% (RR 0.65, 95% confidence interval [CI]: 0.53–0.79) and 33% (RR 0.64, 95% CI: 0.51–0.81) among confirmed cases. Eighty‐four percent of symptomatic cases had fever (mean duration: 1.7 ± 1.0.8 days), 40.5% had cough, and 16.4% had transient leukopenia. Three hundred and seven (81.6%) had an epidemiological exposure in household (69.1%), school (21.8%), and residential area (8.8%).

**Conclusion:**

The surge of pediatric COVID‐19 cases and multiple transmission model reflect wide dissemination of Omicron variant in the community. Asymptomatic infection is common among Omicron‐infected children. COVID‐19 vaccination can offer some protection against symptomatic infection and febrile disease.

## 1 INTRODUCTION

The COVID‐19 pandemic has caused devastation to the world's population and public health crisis. SARS‐CoV‐2 infection in most pediatric cases is mild as compared to adults, and the direct effect on child health is limited.^1^ However, the indirect impacts on medical care, education, and mental health of pediatric patients are considerable owing to lockdown, disruption of essential health service delivery, prolonged school closure, and isolation.^2^, ^3^ The continuous genetic evolution of SARS‐CoV‐2 virus results in the emergence of multiple new variants of concern (VOCs), which are associated with enhanced transmissibility or increased virulence and immune escape.^4^ The Omicron variant, detected in November 2021 and almost replaced Delta variant by the end of January 2022, has led to the fifth global wave of COVID‐19 epidemic.^5^ The significant rise of pediatric infection was reported in the United States with children aged <18 years, representing 17.0–19.0% of all cases during the Omicron period since late December 2021.^6^, ^7^

Pediatric COVID‐19 cases only accounted for a small proportion of infection in the early stages of the COVID‐19 pandemic when many countries implemented non‐pharmaceutical interventions and strict containment measures.^8^, ^9^, ^10^, ^11^, ^12^ However, the incidence rate of COVID‐19 in children showed a rising trend in the epidemic countries when public health measures were lifted.^12^, ^13^ After the large‐scale epidemic in early 2020, China implemented the “dynamic zero” prevention and control strategy to deal with the small‐scale outbreak of SARS‐CoV‐2 variants. Massive COVID‐19 vaccination campaign was launched nationwide in 2021. Inactivated SARS‐CoV‐2 vaccine BBIBP‐CorV (Sinopharm) and CoronaVac (Sinovac) were approved for emergency use in children 3–17 years on June 2021, and COVID‐19 vaccination program was initiated in pediatric population since late July 2021 across China. From May 2020, local pediatric COVID‐19 infection linked to sporadic and cluster transmission were occasionally reported in China until the community outbreak of Omicron variant appeared in Hong Kong Special Administrative Region since January 6, 2022, and subsequently in Shanghai since early March 2020.^14^ Omicron BA2.2 variant spread rapidly in Shanghai by the end of March and led to a surge of pediatric COVID‐19 cases citywide. In this study, we aimed to describe epidemiological and clinical characteristics of the pediatric Omicron infection in Shanghai during the early stage of the outbreak in March 2022.

## 2 METHODS

We collected data of local COVID‐19 cases <18 years of age, who were notified in Shanghai and admitted to the exclusively designated hospital for isolation, observation, or treatment from March 7 to March 31. Prior to March 28 when mass polymerase chain reaction (PCR) testing had not been implemented among citywide population in Shanghai, all pediatric COVID‐19 cases were detected through epidemiological investigation and febrile symptom‐based screening. All confirmed pediatric cases were referred to the designated hospital for treatment and isolation. After a large number of cases were detected since the implementation of mass screening test from March 28 onwards, most of asymptomatic and mild pediatric cases aged 5–17 years were managed by the community isolation facilities since March 29. All confirmed cases irrespective of symptoms were required for isolation in hospitals or community facilities until their cycle threshold (Ct) value for the viral nucleic acid was greater than 35 on PCR test for the two consecutive respiratory samples taken 24 h apart.^15^

### 2.1 Case definition and classification

All COVID‐19 cases were confirmed by the Shanghai Municipal Center for Disease Control and Prevention (CDC) reference laboratory using real‐time RT‐PCR commercial kit. The Ct value <40 was defined as a positive nucleic acid amplification test. The COVID‐19 cases were classified as asymptomatic and symptomatic cases. Symptomatic cases were further classified as mild, moderate, and severe cases. An asymptomatic case is defined as a person with a positive nucleic acid test but without any clinical symptom of COVID‐19. A confirmed symptomatic case is defined as a person presenting clinical signs and symptoms of COVID‐19. COVID‐19 disease severity classification is based on the World Health Organization (WHO) guidance.^16^ Pneumonia was diagnosed based on clinical signs (fever and/or cough accompanying with one of the following signs: moist rales on auscultation, difficulty breathing/dyspnea, fast breathing, and chest indrawing) and radiological findings compatible with pneumonia.

### 2.2 Data collection

Data were collected via a face‐to‐face interview with parents or teenagers and electronic medical chart, including demographic information, epidemiological exposure setting, COVID‐19 vaccination status on dose and date, clinical symptoms, laboratory findings and chest imaging if examined, treatment, and outcome. Informed consent from patients' parents or their grandparents was not required because all data were de‐identified, which is approved by the ethics committee of Children's Hospital of Fudan University.

### 2.3 Statistical analysis

Data were entered in an excel spreadsheet (Microsoft Office 2016, Redmond, Washington) for analysis. The statistical analysis was performed using spss (IBM Statistics 23.0). Categorical variables are described as counts and percentage. Numeric variables with normal distribution were summarized as mean ± standard deviation. Median (interquartile range [IQR]) was used for skewed data. Differences between groups are compared using Mann–Whitney U test and Student's t test as appropriate. A difference with P < 0.05 is considered to be statistically significant. Relative risks (RRs) were calculated to explore the effect of full vaccination (two doses) and partial vaccination (one dose) on the symptomatic infection and febrile disease.

## 3 RESULTS

### 3.1 Demographic characteristics

**FIGURE 1:**
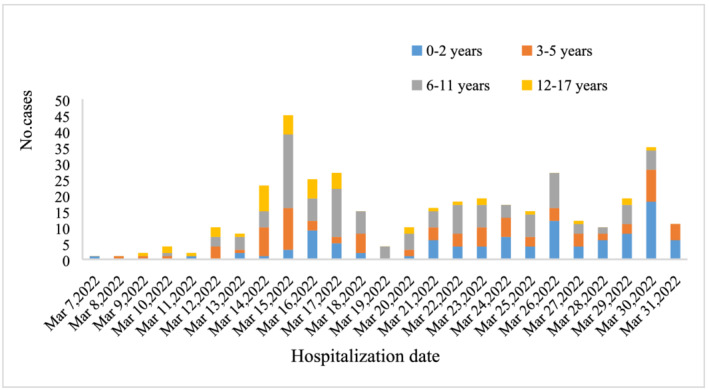
Daily COVID‐19 cases referred to the designated hospital for children aged <18 years from March 1 to March 31, 2022

The local pediatric cases were first detected on March 7, 2022, and the number of cases increased remarkably from March 14 onwards (as shown in Figure 1). As of March 31, a total of 376 pediatric cases of COVID‐19 were referred to the exclusively designated hospital. The ratio of male to female was 1.1 (male 206/376 [54.8%] vs. female 170/376 [45.2%]). The 376 cases were aged 11 days to 17 years with the median age of 5.0 (IQR: 2, 9) years and the mean age of 6.0 ± 4.2 years: 28 (7.4%) cases in age group <1 year, 76 (20.2%) cases in age group 1–2 years, 94 (25.0%) cases in age group 3–5 years, 134 (35.6%) cases in age group 6–11 years, and 44 (11.7%) cases in age group ≥12 years.

### 3.2 Epidemiological exposure

**FIGURE 2:**
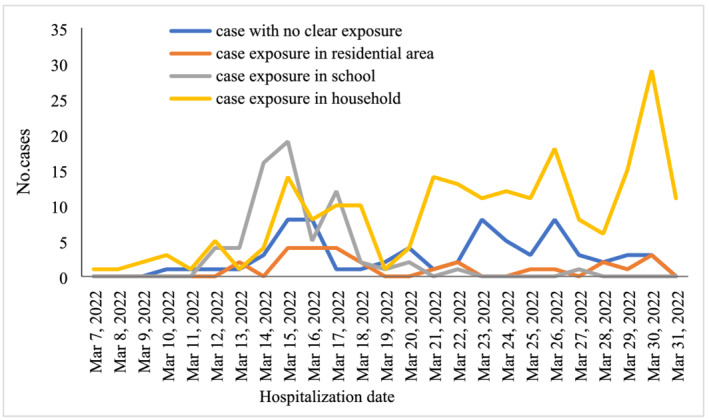
Model of epidemiological exposure over time among pediatric COVID‐19 cases

Three hundred and seven (81.6%) cases had a clear history of COVID‐19 exposure, of whom 213 (69.1%) had a close contact with confirmed adult cases in household, 67 (21.8%) had a close contact with confirmed pediatric cases in the school, and 27 (8.8%) had an epidemiological linkage to residential area where cluster cases of COVID‐19 were reported. As shown in Figure 2, the first child case acquired infection in family; soon after, child cases linked to possible community transmission were found, who had no history of clear exposure.

### 3.3 Vaccination status

A total of 126 children had received at least one dose of an inactivated COVID‐19 vaccine, accounting for 33.5% of the total 376 pediatric cases and 46.3% of the 272 pediatric cases aged ≥3 years eligible for COVID‐19 vaccination. Of the 272 vaccine‐eligible children, 146 (53.6%) were unvaccinated, 110 (40.4%) had received two doses, and 16 (4.0%) had received one dose. Among the 94 pre‐school children aged 3–5 years, the proportions of one dose and two doses of COVID‐19 vaccination were 3.2% (3/94) and 5.3% (5/94), respectively. Among the 178 school children aged 6–17 years, the proportions of one dose and two doses of COVID‐19 vaccination were 7.3% (13/178) and 59.0% (105/178), respectively. Overall, the interval between vaccination and breakthrough infection ranged from 16 days to 7 months (median: 3.5 [IQR: 3, 4.5] months).

As shown in Table 1, 22.8% (57/250) of unvaccinated cases were asymptomatic, whereas 50.0% (55/110) of fully vaccinated cases were asymptomatic (P < 0.001); 65.2% (163/250) of unvaccinated cases were febrile, whereas 41.8% (46/110) of fully vaccinated cases were febrile (P < 0.001). The full COVID‐19 vaccination reduced the RR of symptomatic infection by 35% (0.65, 95% confidence interval [CI]: 0.53–0.79) and the risk of febrile disease by 36% (RR 0.64, 95% CI: 0.51–0.81) in children 0–17 years and by 29% (RR 0.71, 95% CI: 0.57–0.88) and 29% (RR 0.71, 95% CI: 0.55–0.92) in children 3–17 years eligible for COVID‐19 vaccine (Table 2). However, the benefit of partial vaccination (one dose) did not yield statistical significance (Table 2).

**TABLE 1:** Clinical characteristics of SARS‐CoV‐2 virus infection by age group

Clinical characteristics	Total (n = 376)	Age group (years)			P value	Vaccination status		P value
		<3 (n = 104)	3–5 (n = 94)	6–17 (n = 178)		Unvaccinated (n = 250)	2‐dose vaccination (n = 110)	
Asymptomatic cases, n (%)	119 (31.6%)	14 (13.5%)	29 (30.9%)	76 (42.7%)	<0.001	57 (22.8%)	55 (50.0%)	<0.001
Symptomatic cases, n (%)	257 (68.4%)	90 (86.5%)	65 (69.1%)	102 (57.3%)	<0.001	193 (77.2%)	55 (50.0%)	<0.001
Febrile cases, n (%)	216 (57.4%)	44 (42.3%)	59 (62.8%)	80 (44.9%)	0.006	163 (65.2%)	46 (41.8%)	<0.001
Fever spike (°C), mean ± SD	38.9 ± 0.6	39.0 ± 0.7	39.0 ± 0.6	38.8 ± 0.6	0.064	38.9 ± 0.6	38.8 ± 0.7	0.27
Fever duration (days), mean ± SD	1.7 ± 0.8	1.7 ± 0.9	1.6 ± 0.6	1.8 ± 0.8	0.45	1.6 ± 0.8	1.9 ± 0.9	0.19

**TABLE 2:** Clinical characteristics of SARS‐CoV‐2 infection according to COVID‐19 vaccination status

Vaccination status by age group (years)	Symptomatic case, n (%)	Relative risk (95% CI)	Febrile cases, n (%)	Relative risk (95% CI)
0–17 (n = 376)				
Unvaccinated (n = 250)	193 (77.2%)	Ref	163 (65.2%)	Ref
1 dose (n = 16)	9 (56.3%)	0.73 (0.47–1.13)	7 (43.8%)	0.67 (0.38–1.18)
2 doses (n = 110)	55 (50.0%)	0.65 (0.53–0.79)	46 (41.8%)	0.64 (0.51–0.81)
3–17 (n = 272)				
Unvaccinated (n = 146)	103 (70.5%)	Ref	86 (58.9%)	Ref
1 dose (n = 16)	9 (56.3%)	0.80 (0.51–1.24)	7 (43.8%)	0.74 (0.42–1.32)
2 doses (n = 110)	55 (50.0%)	0.71 (0.57–0.88)	46 (41.8%)	0.71 (0.55–0.92)

Abbreviations: CI, confidence interval; Ref, reference group.

### 3.4 Clinical manifestation and course of disease

Of the 376 cases, 257 (68.4%) manifested with symptoms and 119 (31.6%) were asymptomatic before and during hospitalization. Of the 257 symptomatic cases, 216 (84.0%) experienced fever (axillary temperature >37.5°C) with a mean fever spike of 38.9 ± 0.6°C (range: 37.6–41°C) and a mean fever duration of 1.7 ± 1.0.8 days (range: 0.5–4 days), 104 (40.5%) had cough, 28 (10.9%) self‐reported sore throat, 13 (5.1%) self‐reported stuffy nose, 6 (2.3%) had runny nose, 11 (4.3%) had nausea or vomiting or diarrhea, and 2 (0.8%) self‐reported transient loss of taste and smell. No severe case was found. Six (1.6%) cases had comorbidity including brain tumor, febrile seizure, psychomotor retardation, hemophilia, Henoch‐Schönlein purpura, and cardiac arrhythmia. As shown in Table 1, symptomatic infection was significantly frequently seen in the age group <3 years than in the age groups 3–5 years (P = 0.003) and 6–17 years (P = 0.000). Fever was significantly frequently seen in the age group 3–5 years than in the age groups <3 years (P = 0.000) and 6–17 years (P = 0.005).

Twenty‐five cases had chest computed tomography (CT) scan performed due to fever >38.5°C lasting for 3 days or cough worsening after admission or routine examination prior to the referral. The chest images showed patchy infiltrates or ground‐glass opacity in four cases and right lung lobar pneumonia in an 8‐year‐old boy who was co‐infected with *Mycoplasma pneumoniae*. Of the 225 cases who had complete peripheral blood cell count tested, 37 (16.4%) had white blood cell (WBC) count <4 × 10^9^/L, 173 (76.9%) had WBC count 4–9 × 10^9^/L, 13 (5.8%) had WBC count 10–14 × 10^9^/L, and 2 (0.9%) had WBC count ≥15 × 10^9^/L. The WBC count ranged from 1.9 × 10^9^/L to 15.5 × 10^9^/L. No thrombopenia was observed. Of the 187 cases who had peripheral blood C‐reactive protein (CRP) tested, 178 (95.2%) had CRP < 8 mg/L, 8 (4.3%) had CRP > 8 mg/L (range: 8.8–35.8 mg/L), and 1 (0.5%) had CRP 56 mg/L who had co‐infection with *M. pneumoniae* and developed typical lobar pneumonia in the right lung. Of the 196 cases who had serum biochemical markers, 8 (4.1%) showed slightly elevated liver enzyme.

For symptomatic cases, Ibuprofen and or Chinese traditional medicines were prescribed depending on individualized condition and medication compliance. Only one case with lobar pneumonia who had a co‐infection with *M. pneumoniae* was prescribed with antibiotics. All cases were discharged when the Ct value of the SARS‐CoV‐2 nucleic acid reached >35. The average duration of Ct value of the nucleic acid of SARS‐CoV‐2 virus was >35 because admission was 11.7 ± 3.7 days (range: 3–25 days; symptomatic vs. asymptomatic: 11.7 ± 3.6 vs. 11.7 ± 3.9, P = 0.064).

## 4 DISCUSSION

To our knowledge, our study is the first study to present the epidemiological and clinical profiles of Omicron variant infection in children in Shanghai during the early phase of outbreak. All pediatric COVID‐19 cases were mild (68.4%) or asymptomatic (31.6%), whereas a few of severe pediatric cases were reported during the early stage of COVID‐19 outbreak in Wuhan in 2020.^17^ Moreover, the proportion of asymptomatic cases was 2 times of that seen in the initial Wuhan outbreak. Part explanation was that the high coverage of COVID‐19 vaccination has lowered the risk of severe Omicron‐associated diseases. The mass COVID‐19 vaccination rollout among children 3–17 years started between mid‐August 2021 and December 2021 in Shanghai. The estimated coverage of full COVID‐19 vaccination was more than 70% in children 3–17 years by the end of March in 2022. The median time of Omicron infection after COVID‐19 vaccination was 3.5 months in this case cohort. Observational studies in countries with high levels of population immunity generated by natural infection or vaccine have shown receipt of two doses of COVID‐19 vaccines, and a booster dose can offer protection against symptomatic and severe Omicron infection within a short‐term period of vaccination.^4^, ^18^, ^19^, ^20^, ^21^ A recent system review reported that vaccine effectiveness of primary series COVID‐19 vaccines against severe Omicron infection mostly sustained at 6 months after full vaccination.^22^

Current evidences consistently showed a reduction in neutralizing antibody against Omicron in serum of convalescent or vaccinated individuals, resulting in Omicron's immune escape potential against vaccine‐ and infection‐induced immunity.^4^, ^23^, ^24^ However, two recent studies based on real‐world observation among children showed the modest effectiveness for COVID‐19 vaccine against Omicron infection.^25^, ^26^ We found that receipt of two‐dose inactivated COVID‐19 vaccine within 17 days to 7 months after fully primary vaccination potentially reduced the risk of symptomatic Omicron infection by 31% and febrile disease by 59% in children. We could not estimate vaccine protection against severe infection because no severe COVID‐19 cases were found in our study. The current evidence showed vaccine effectiveness waning over time of the primary series against COVID‐19 for the studied vaccines.^4^ However, the vaccine effectiveness against Omicron infection and disease can be restored and increase to >40% to 80% within a short follow‐up time after a third booster dose in studies from five countries (United Kingdom, Denmark, Canada, South Africa, and United States).^4^ Ten cases of reinfection with Omicron variant were identified within 23 to 87 days of a previous Delta infection reported in the United States, and most were pediatric cases.^26^ Thus, it is necessary to improve the acceptance of full COVID‐19 vaccination among eligible children and adolescents in response to Omicron outbreak. So far, a third booster dose of COVID‐19 vaccine has been recommended for use in adults but not in children in China. In light of the field findings, a booster dose should also be recommended for eligible children.

Most of pediatric cases had a clear epidemiological exposure in household, school, and residential area. We noticed that 88.3% of confirmed pediatric cases were school children 6–11 years, pre‐school children 3–5 years, and home‐cared children <3 years, suggesting that the cluster transmission of Omicron infection mainly occurred in elementary school, kindergarten, and household during the early epidemic wave. However, 80–90% of confirmed child cases were family cluster cases, whereas community and school transmission was unusual in children during the 2020 outbreak of COVID‐19 in China.^17^, ^27^ Meanwhile, we found that 18.4% of pediatric cases had no determined exposure, suggesting that silent transmission had occurred in the community during the early epidemic wave.

We observed that most of pediatric cases with symptomatic Omicron infection manifest with fever. However, fever was less commonly seen in pediatric COVID‐19 cases reported in China (58%) and the United States (56%) during the first wave of pandemic in 2020.^10^, ^17^ Fever is helpful for early recognition and diagnosis of COVID‐19 because parents always worry about the febrile child and seek medical care. The febrile course of Omicron infection is brief with a mean fever duration of 1.7 days, significantly shorter than fever duration seen in influenza (4 days).^28^ The febrile duration could potentially help to differentiate COVID‐19 from influenza in children if the epidemics of COVID‐19 and influenza overlap.

The potential role in transmission for most asymptomatic and mild child cases should not be neglected. A study showed that symptomatic and asymptomatic children can carry high quantities of live SARS‐CoV‐2, creating a potential reservoir for transmission.^29^ Vaccinees with mild or asymptomatic Omicron infection shed infectious virus 6–9 days after onset or diagnosis, even after symptom resolution.^30^ In fact, asymptomatic infection in children was underestimated in the early stage of outbreak because mass screening of COVID‐19 cases had not been carried out before March 28. After citywide large‐scale screening, notifiable asymptomatic cases accounted for 90% more or less in April. Asymptomatic infection was much more common in vaccinated children than in unvaccinated children (50% vs. 22.8%). Vaccination can offer some protection against symptomatic infection and febrile disease. On the other hand, the role of asymptomatic children play in viral transmission is of attention during outbreak. High prevalence of asymptomatic infection is likely a major factor in the widespread of the Omicron variant among population.

In summary, SARS‐CoV‐2 infection is mild and subtle among children in Shanghai with the high coverage of COVID‐19 vaccine during the early stage of Omicron outbreak. The full COVID‐19 vaccination can offer partial protection against symptomatic COVID‐19. Ongoing Omicron epidemic will increase the risk of exposure among children with underlying medical conditions, who are usually unvaccinated. Therefore, severe COVID‐19 infection is anticipated to be encountered in children. Non‐pharmaceutical interventions in combination with vaccination strategies are critical to prevent infection and severe disease and to mitigate the impact of COVID‐19 in pediatric population.

## CONFLICTS OF INTEREST

The authors declare that there are no conflicts of interest in relation to this work.

## AUTHOR CONTRIBUTIONS

**Xiangshi Wang:** Conceptualization; data curation; investigation; methodology. **Hailing Chang:** Data curation; formal analysis; investigation; methodology. **He Tian:** Data curation; formal analysis; investigation; methodology. **Yanfeng Zhu:** Data curation; investigation; methodology. **Jingjing Li:** Data curation; investigation; methodology. **Zhongqiu Wei:** Data curation; investigation; methodology. **Yixue Wang:** Data curation; investigation; methodology. **Aimei Xia:** Investigation; methodology; resources. **Yanling Ge:** Data curation; investigation; methodology. **Gongbao Liu:** Investigation; methodology; resources. **Jiehao Cai:** Data curation; investigation; methodology. **Xiaowen Zhai:** Data curation; investigation; methodology. **Qirong Zhu:** Data curation; investigation; methodology; resources. **Mei Zeng:** Conceptualization; data curation; formal analysis; funding acquisition; investigation; methodology; project administration; resources; supervision.

### PEER REVIEW

The peer review history for this article is available at https://publons.com/publon/10.1111/irv.13044.

## Data Availability

The data that support the findings of this study are available from the corresponding author upon reasonable request.
